# Antibiotic Prophylaxis for Presumptive Group B Streptococcal Infection in Preterm Premature Rupture of the Membranes: Effect on Neonatal and Maternal Infectious Morbidity

**DOI:** 10.1155/S1064744996000634

**Published:** 1996

**Authors:** Wayne B. Kramer, George R. Saade, Michael Belfort, Joanne Samora-Mata, Tony Wen, Kenneth J. Moise

**Affiliations:** 1 Division of Maternal-Fetal Medicine Department of Obstetrics and Gynecology Baylor College of Medicine Houston TX USA; 2 Department of Pharmacology Baylor College of Medicine Houston TX USA; 3 Department of Anesthesiology Baylor College of Medicine Houston TX USA; 4 Department of Medicine Baylor College of Medicine Houston TX USA; 5 Department of Obstetrics, Gynecology, and Reproductive Medicine University of Maryland Medical Center 405 West Redwood Street 4th Floor Baltimore MD 21201-1703 USA

## Abstract

*Objective:* The purpose of this study was to determine if the prevalence of neonatal and maternal infectious morbidity in patients with preterm premature rupture of membranes (PROM) who received ampicillin prophylaxis for presemptive group B streptococcal colonization is increased compared to those who received no prophylaxis.

*Methods:* The charts of all patients with preterm PROM who delivered between January 1988 and December 1993 were retrospectively reviewed. The routine use of ampicillin prophylaxis was initiated in January 1991. Patients with singleton gestations were included in the analysis only if chorioamnionitis was excluded on admission. Variables used in the final analysis included gestational age at the time of preterm PROM, gestational age at delivery, duration of rupture of membranes, birth weight, method of delivery, use of steroids, tocolytics, or antibiotics for group B streptococcus prophylaxis, neonatal sepsis, neonatal mortality, and postpartum endomyometritis. Data were analyzed using Student's t-test, chi-square test, Fisher's exact test, and stepwise logistic regression analysis to evaluate the effect of chemoprophylaxis for group B streptococcus on the incidence of neonatal sepsis and maternal postpartum endomyometritis. A two-tailed *P* < 0.05 was used to denote statistical significance.

*Results:* The charts of 206 patients were reviewed; 146 patients received ampicillin for group B streptococcal prophylaxis and 60 patients did not. There was a significantly higher incidence of postpartum endomyometritis among the patients who received ampicillin (62% vs. 22%; *P* < 0.01). The association between postpartum endomyometritis and chemoprophylaxis remained significant even after controlling for other confounding variables. There was no significant difference in the incidence of neonatal sepsis (5% vs. 7%; *P* = 0.7) or death (5% vs. 3%; *P* = 0.9) between both groups.

*Conclusions:* Group B streptococcal prophylaxis with a short course of intravenous ampicillin increases the risk of postpartum endomyometritis in patients with premature PROM.


Infectious Diseases in Obstetrics and Gynecology 4:313–318 (1996)
© 1997 Wiley-Liss, Inc.

### Antibiotic Prophylaxis for Presumptive Group B Streptococcal Infection in Preterm Premature Rupture of the Membranes: Effect on Neonatal and Maternal Infectious Morbidity

Wayne B. Kramer,[1,*] George R. Saade,[1,2] Michael Belfort,[1,3,4] Joanne Samora-Mata,[1] Tony Wen,[1] and Kenneth J. Moise, Jr.[1]

[1]*Division of Maternal-Fetal Medicine, Department of Obstetrics and Gynecology, Baylor College of Medicine, Houston, TX*
[2]*Department of Pharmacology, Baylor College of Medicine, Houston, TX*
[3]*Department of Anesthesiology, Baylor College of Medicine, Houston, TX*
[4]*Department of Medicine, Baylor College of Medicine, Houston, TX*

### Abstract

*Objective:* The purpose of this study was to determine if the prevalence of neonatal and maternal infectious morbidity in patients with preterm premature rupture of membranes (PROM) who received ampicillin prophylaxis for presemptive group B streptococcal colonization is increased compared to those who received no prophylaxis.

*Methods:* The charts of all patients with preterm PROM who delivered between January 1988 and December 1993 were retrospectively reviewed. The routine use of ampicillin prophylaxis was initiated in January 1991. Patients with singleton gestations were included in the analysis only if chorioamnionitis was excluded on admission. Variables used in the final analysis included gestational age at the time of preterm PROM, gestational age at delivery, duration of rupture of membranes, birth weight, method of delivery, use of steroids, tocolytics, or antibiotics for group B streptococcus prophylaxis, neonatal sepsis, neonatal mortality, and postpartum endomyometritis. Data were analyzed using Student's t-test, chi-square test, Fisher's exact test, and stepwise logistic regression analysis to evaluate the effect of chemoprophylaxis for group B streptococcus on the incidence of neonatal sepsis and maternal postpartum endomyometritis. A two-tailed $P < 0.05$ was used to denote statistical significance.

*Results:* The charts of 206 patients were reviewed; 146 patients received ampicillin for group B streptococcal prophylaxis and 60 patients did not. There was a significantly higher incidence of postpartum endomyometritis among the patients who received ampicillin (62% vs. 22%; $P < 0.01$). The association between postpartum endomyometritis and chemoprophylaxis remained significant even after controlling for other confounding variables. There was no significant difference in the incidence of neonatal sepsis (5% vs. 7%; $P = 0.7$) or death (5% vs. 3%; $P = 0.9$) between both groups.

*Conclusions:* Group B streptococcal prophylaxis with a short course of intravenous ampicillin increases the risk of postpartum endomyometritis in patients with premature PROM. Infect. Dis. Obstet. Gynecol. 4:313–318, 1996. © 1997 Wiley-Liss, Inc.

Key words ampicillin; endomyometritis; neonatal sepsis

*Correspondence to: Dr. Wayne B. Kramer, Department of Obstetrics, Gynecology, and Reproductive Medicine, University of Maryland Medical Center, 405 West Redwood Street, 4th Floor, Baltimore, MD 21201-1703.

Received 14 September 1995
Accepted 14 January 1997

Clinical Study

The goal of the physician managing a patient with preterm premature rupture of the membranes (PROM) is to minimize the risks of both respiratory distress syndrome and maternal and neonatal infection. The etiology of preterm PROM is, however, poorly understood. There is evidence suggesting that an inflammatory process from either a local ascending vaginal or intra-amniotic bacterial infection may weaken the membranes, thus leading to membrane rupture.[1] This has led to numerous trials utilizing antibiotic prophylaxis in patients with preterm PROM.[2–6] However, there is no consensus in the literature regarding the choice of antibiotic or the duration of therapy that should be undertaken.

Group B streptococcus has been shown to be a risk factor for the development of preterm PROM, neonatal infection, and postpartum febrile morbidity.[1,7,8] Numerous studies have shown a reduction in the incidence of early-onset neonatal group B streptococcal disease after maternal antibiotic chemoprophylaxis.[9–12] These studies have led both the American College of Obstetricians and Gynecologists (ACOG)[13] and the American Academy of Pediatrics (AAP)[14] to recommend treatment with either penicillin or ampicillin in patients with unknown status for group B streptococcal colonization who are intrapartum with preterm PROM. Since in most centers the majority of patients would fall into this category, these recommendations would lead to the use of anbibiotics in a large number of patients with preterm PROM. Neither college, however, has defined the duration of therapy in a patient who remains undelivered. The purpose of this study was to determine if the prevalence of neonatal and maternal infectious morbidity in patients with preterm PROM who received ampicillin prophylaxis for presumptive group B streptococcal colonization is increased compared to those who received no prophylaxis.

### SUBJECTS AND METHODS

The records of all patients with preterm PROM admitted prior to 37 weeks of gestation to the Baylor Perinatal Service at St. Luke's Episcopal Hospital between January 1989 and December 1993 were reviewed. These patients were all referred to the Baylor Perinatal Service by private physicians and copies of their records were maintained both at St. Luke's Episcopal Hospital and at the Baylor Perinatal Service offices. PROM was documented by the presence of obvious pooling or fern and nitrazine-positive fluid in the posterior fornix on sterile speculum examination. Patients with singleton gestations were included in the analysis only if chorioamnionitis was excluded on admission (oral temperature ≤100.4°F and no evidence of uterine tenderness, fetal tachycardia, or foul smelling vaginal discharge), and the patient was confined to conservative management without active intervention. Conservative management consisted of bed rest, twice weekly biophysical profiles, and external fetal heart rate monitoring 3 times daily. The best estimate of gestational age was determined on admission using menstrual history, prenatal records, and ultrasound criteria.

A policy of routine chemoprophylaxis for group B streptococcus was initiated in January 1991. Prior to this time, chemoprophylaxis was administered based on the individual physician's index of suspicion or findings on Gram stain of cervical culture. After this time, all patients with preterm PROM managed conservatively were given intravenous ampicillin (2 g bolus, followed by 1 g every 4 h) until the result of the anogenital cultures for group B streptococcus became available (usually within 48–72 h). All cultures were obtained via culturette from the cervix, lower third of the vagina, and the anus. The culture swabs were transported to the laboratory where they were inoculated within 2 h on both blood agar plates and Todd-Hewitt broth. These were then incubated for 18–24 h. If the culture was positive, the patient was given amoxicillin 500 mg orally every 8 h to complete a total of 10 days of antibiotic therapy. Intravenous antibiotics were discontinued if the culture returned negative. Patients allergic to penicillin were given cefoxitin 1 g intravenously every 6 h. Tocolytics were used according to the discretion of the treating physician. Antenatal steroids to enhance fetal lung maturity were not routinely used during this time period. Cervical evaluations were performed visually by sterile speculum examination, while digital examinations were reserved for patients in active labor. Labor was induced if a non-reassuring fetal heart rate pattern was documented on the electronic fetal heart monitoring, intra-amniotic infection was evident, or patients attained a gestational age of 36 weeks. Diagnosis of intra-amniotic infection was based on maternal fever, maternal or fetal tachycardia, uterine tenderness, foul odor of amniotic fluid, and leukocytosis. All patients were restarted on ampicillin intravenously once labor was induced.

Neonatal sepsis was defined as positive blood cultures. The patient was considered to have developed postpartum endomyometritis if the primary physican documented uterine tenderness to clinical examination and leukocytosis in conjunction with an oral temperature ≥100.4°F on at least 2 occasions 6 h apart. Endometrial and anogenital cultures were not routinely performed on patients developing endomyometritis.

Variables used in the final analysis included gestational age at the time of preterm PROM ($GA_{PROM}$), gestational age at delivery ($GA_{DEL}$), length of the latency period (defined as the interval between membrane rupture and delivery, $PROM_{LAT}$), birth weight (BW), delivery method (vaginal vs. cesarean section), use of antibiotics for group B streptococcal prophylaxis, steroids or tocolytics, neonatal sepsis, neonatal mortality, and postpartum endomyometritis.

The data were analyzed using Student's chisquare test, or Fisher's exact test as appropriate. The D'Agostino test was used to check for normalcy of distribution of the data sets. A stepwise logistic regression analysis was used to evaluate the effect of group B streptococcal chemoprophylaxis on the incidence of neonatal sepsis and maternal postpartum endomyometritis after controlling for other confounding variables. A two-tailed $P < 0.05$ was used to denote statistical significance.

### RESULTS

The charts of 206 patients with preterm PROM were reviewed. A total of 146 patients received chemoprophylaxis; 60 patients did not receive prophylaxis. Seven patients received chemoprophylaxis prior to January 1991 with only 1 developing postpartum endomyometritis. No patients received cefoxitin. There was a 25% prevalence of group B streptococcus at initial culture. Table 1 compares the demographic characteristics of the two groups. There was no significant difference in the incidence of neonatal sepsis (5% vs. 7%; $P = 0.7$) or death (5% vs. 3%; $P = 0.9$) between patients who received antibiotic prophylaxis and those who did not. There was a significantly higher incidence of postpartum endomyometritis in patients who received prophylaxis (62% vs. 22%; $P < 0.01$). The latency period was similar in both groups (9 vs. 7 days; $P = 0.3$).

**TABLE 1. Demographic data[a]**

| | Ampicillin (n = 146) | No ampicillin (n = 60) | *P* |
|---|---|---|---|
| $GA_{PROM}$ (weeks) | 29.9 ± 3.2 | 30.7 ± 3.2 | NS |
| $GA_{DEL}$ (weeks) | 31.2 ± 2.9 | 31.7 ± 2.9 | NS |
| $PROM_{LAT}$ (days) | 9 ± 12 | 7 ± 10 | NS |
| BW (g) | 1,694 ± 557 | 1,836 ± 586 | NS |
| Vaginal delivery | 95 (65) | 44 (73) | NS |
| Received steroids | 12 (8) | 3 (5) | NS |
| Received tocolytics | 77 (53) | 33 (55) | NS |
| Neonatal sepsis | 8 (5) | 4 (7) | NS |
| Neonatal death | 7 (5) | 2 (3) | NS |
| Postpartum endomyometritis | 90 (62) | 13 (22) | <0.01 |

[a]Mean ± standard deviation or number (%). NS, not significant.

Table 2 summarizes the univariate analysis of risk factors for developing neonatal sepsis. Neonates who developed sepsis had a significantly lower birth weight and gestational age at PROM and delivery compared to those who did not. The latency period and the number of patients who delivered vaginally, received antibiotic prophylaxis, tocolytics, or steroids were not significantly different between the two groups. Group B streptococcus sensitive to ampicillin was isolated from 4 neonates developing neonatal sepsis (2 in each group). *Escherichia coli* and *Staphylococcus aureus*, which were resistant to ampicillin, were isolated among the remainder.

Patients diagnosed with postpartum endomyometritis had a lower gestational age at delivery and birth weight (Table 3). They were significantly more likely to have been delivered by cesarean section and to have received group B streptococcal prophylaxis. The latency period and the number of patients receiving tocolytics or steroids, however, were not significantly different between those who developed postpartum endomyometritis and those who did not.

Of the patients with vaginal delivery, endomyometritis developed in 47% with vs. 7% without antibiotic prophylaxis ($P < 0.01$). Of the patients delivering by cesarean section, endomyometritis developed in 88% with vs. 63% without antibiotic prophylaxis ($P < 0.04$). However, the association between postpartum endomyometritis and ampicillin remained significant even after controlling for gestational age at delivery, birth weight, adminis-

TABLE 2. Univariate analysis of risk factors for neonatal sepsis[a]

| | Sepsis (n = 12) | No sepsis (n = 194) | Odds ratio | 95% CI | *P* |
|---|---|---|---|---|---|
| $GA_{PROM}$ (weeks) | 27.9 ± 3.3 | 30.3 ± 3.1 | — | 0.5–4.2 | 0.03 |
| $GA_{DEL}$ (weeks) | 29.4 ± 2.8 | 31.5 ± 2.9 | — | 0.4–3.8 | 0.02 |
| $PROM_{LAT}$ (days) | 11 ± 12 | 8 ± 12 | — | −1.3–0.7 | NS |
| BW (g) | 1,377 ± 459 | 1,758 ± 568 | — | 50.3–710.1 | 0.02 |
| Vaginal delivery | 6 (50) | 133 (69) | 0.50 | 0.1–1.7 | NS |
| Received steroids | 1 (8) | 14 (7) | 1.20 | 0.1–10.0 | NS |
| Received tocolytics | 5 (42) | 105 (54) | 0.60 | 0.2–2.2 | NS |
| Received ampicillin | 8 (67) | 138 (71) | 0.80 | 0.2–3.4 | NS |

[a]Mean ± standard deviation or number (%). NS, not significant; CI, confidence interval.

TABLE 3. Univariate analysis of risk factors for postpartum endomyometritis[a]

| | Endomyometritis (n = 103) | No endomyometritis (n = 103) | Odds ratio | 95% CI | *P* |
|---|---|---|---|---|---|
| $GA_{PROM}$ (weeks) | 29.8 ± 3.0 | 30.5 ± 3.3 | — | 0.2–1.6 | NS |
| $GA_{DEL}$ (weeks) | 30.9 ± 3.1 | 31.9 ± 2.7 | — | −1.8–−0.2 | 0.02 |
| $PROM_{LAT}$ (days) | 8 ± 10 | 10 ± 13 | — | −0.7–0.2 | NS |
| BW (g) | 1,654 ± 595 | 1,816 ± 531 | — | 7.9–317.8 | 0.04 |
| Cesarean section | 55 (53) | 12 (12) | 8.69 | 4.0–19.0 | 0.01 |
| Received steroids | 8 (8) | 7 (7) | 1.15 | 0.4–3.7 | NS |
| Received tocolytics | 62 (60) | 48 (47) | 1.70 | 0.9–3.1 | NS |
| Received ampicillin | 90 (87) | 56 (54) | 5.80 | 2.8–12.5 | 0.01 |

[a]Mean ± standard deviation or number (%). NS, not significant; CI, confidence interval.

tration of steroids or tocolytics, latency period, and cesarean section (Table IV).

### DISCUSSION

Based on the studies demonstrating that antibiotic prophylaxis reduces the incidence of early neonatal group B streptococcal disease,[9–12] we initiated a policy of routine intrapartum prophylaxis for presumptive group B streptococcal colonization with intravenous ampicillin in all patients with preterm PROM in January 1990. Subsequently, both the ACOG[13] and AAP[14] have passed recommendations endorsing this practice. This policy did not result in a reduction in the incidence of blood culture-proven neonatal sepsis. We found, however, that after the change in policy, postpartum endomyometritis was occurring more often. This resulted in concern that the universal use of ampicillin for group B streptococcal prophylaxis may lead to undesired effects on the maternal and fetal bacterial flora.

Prophylactic antibiotic therapy in patients with preterm PROM has not been shown to reduce the prevalence of blood culture-proven neonatal sepsis[3–6] and postpartum endomyometritis.[2–4,6] Amon

TABLE 4. Multivariate analysis of risk factors for postpartum endomyometritis[a]

| | Odds ratio | 95% CI | *P* |
|---|---|---|---|
| $GA_{DEL}$ | 0.90 | 0.7–1.1 | 0.4 |
| BW | 1.00 | 0.9–1.0 | 0.6 |
| Received steroids | 1.20 | 0.3–4.3 | 0.8 |
| Received tocolytics | 2.00 | 1.0–4.1 | <0.05 |
| $PROM_{LAT}$ | 0.80 | 0.6–0.9 | <0.02 |
| Cesarean section | 12.60 | 5.1–30.8 | <0.001 |
| Received ampicillin | 9.30 | 3.8–22.5 | <0.001 |

[a]Factors entered into the regression from top to bottom. CI, confidence interval.

et al.[2] reported a lower prevalence of neonatal infectious complications in patients treated with ampicillin prophylaxis (1/42) vs. placebo (6/36). They also showed a trend toward a decreased prevalence of neonatal and perinatal mortality, and a trend toward an increased prevalence in postpartum endometritis. Johnston and coworkers[5] have demonstrated a lower prevalence of endomyometritis in patients treated with mezlocillin initially and ampicillin until delivery. Conversely, McDuffie et al.[15] have reported a case series of adverse perinatal outcome associated with the development of resistant Enterobacteriaceae after ampicillin or amoxicillin prophylaxis in patients with preterm PROM. In 2 of the 4 cases described, the patients developed postpartum endomyometritis and pelvic vein thrombophlebitis. Prophylactic antibiotics have, however, been shown to prolong the latency period in patients with preterm PROM.[2–6] This has led to fewer infants being admitted to an intensive care nursery and a lower incidence of neonatal respiratory complications.

Abdominal delivery is a predisposing factor to the development of postpartum endomyometritis. Work by Gonik et al.[16] supports the hypothesis that incipient infection at the time of cesarean delivery may limit the effectiveness of antimicrobial prophylaxis and predispose the patient to develop postpartum infection. Watts et al.[17] demonstrated that the isolation of group B streptococcus or *Enterococcus faecalis* from the upper genital tract prior to cesarean delivery was significantly associated with postpartum endomyometritis. They found that group B streptococcus was susceptible to the prophylactic agents used, suggesting that virulence factors other than antibiotic resistance may be important in the development of postpartum infectious morbidity.

We found a higher prevalence of sepsis in the infants of patients who experienced ruptured membranes and delivery earlier in gestation. As suggested by Morales and Lim,[12] inhibition of group B streptococcal infection by amniotic fluid may be more effective with advancing gestational age, therefore affording greater protection to more advanced gestations. Immaturity itself may predispose the neonate to the development of bacterial sepsis. In our study, the latency period between premature PROM and delivery was also prolonged in the neonates who developed sepsis. This may represent a group of patients in whom a short course of ampicillin merely masked an underlying subclinical intra-amniotic infection. The antibiotics may not have achieved adequate, sustained concentrations to eradicate the bacteria, resulting in a prolonged latency period. The effect of a short course of antibiotics on the maternal bacterial flora and possible development of resistant or more virulent strains have not been fully investigated.

Postpartum endomyometritis occurred in 50% of our patients receiving antibiotic prophylaxis. This rate is much higher than the expected 3% after vaginal delivery and 12–51% after cesarean section at term.[4] Antibiotic prophylaxis at time of cesarean delivery has decreased the risk of postpartum endometritis, however, prophylactic failures do occur. After having received a short course of antibiotic prophylaxis the vaginal bacterial flora may be altered, leading to a resistant species of bacteria which then would cause ascending infection after delivery. Even after controlling for route of delivery, gestational age at delivery, birth weight, and latency period, we found that antibiotic prophylaxis predisposes the patient to the development of postpartum infection.

Amstey and Gibbs[18] have proposed that penicillin G with its narrower and more specific spectrum of activity against group B streptococcus is a better choice than ampicillin for prophylaxis. However, further research into how various group B streptococcal virulence factors are modified by antibiotics, the duration of therapy necessary in colonized patients with preterm PROM, and whether resistant bacterial strains will develop is necessary before definitive recommendations are offered.

In summary, a policy of universal treatment with ampicillin for Group B streptococcus prophylaxis resulted in an increased risk of postpartum endomyometritis without a clear reduction in the prevalence of neonatal sepsis.

### REFERENCES

1. Garite TJ: Premature rupture of the membranes. In Creasy RK, Resnik R (eds): Maternal-Fetal Medicine: Principles and Practice. Philadelphia: W.B. Saunders, pp 625–626, 1994.
2. Amon E, Lewis S, Sibai B, et al.: Ampicillin prophylaxis in preterm premature rupture of membranes: A prospective randomized study. Am J Obstet Gynecol 159:539–543, 1988.
3. Christmas JT, Cox SM, Andrews W, et al.: Expectant management of preterm ruptured membranes: Effects of antimicrobial therapy. Obstet Gynecol 80:759–762, 1992.
4. Ernest JM, Givner LB: A prospective, randomized, placebo-controlled trial of penicillin in preterm premature rupture of membranes. Am J Obstet Gynecol 170:516–521, 1994.
5. Johnston MM, Sanchez-Ramos L, Vaughn AJ, Todd MW, Benrubi GI: Antibiotic therapy in premature rupture of the membranes: A randomized, prospective, double-blind trial. Am J Obstet Gynecol 163:743–747, 1990.
6. McGregor JA, French JI, Seu K: Antimicrobial therapy in preterm rupture of membranes: Results of a prospective, double blind, placebo-controlled trial of erythromycin. Am J Obstet Gynecol 165:743–747, 1991.

7. Gibbs RS, Sweet RL: Maternal and fetal infections: Clinical disorders. In Creasy RK, Resnik R (eds): Maternal-Fetal Medicine: Principles and Practice. Philadelphia: W.B. Saunders, pp 646–656, 1994.
8. Newton ER, Clark M: Group B streptococcus and preterm rupture of membranes. Obstet Gynecol 71:198–202, 1988.
9. Matorras R, Garcia-Parea A, Omenaca F, Diez-Enciso D, Madero R, Usandizaga JA: Intrapartum chemoprophylaxis of early onset group B streptococcal disease. Eur J Obstet Gynecol Reprod Biol 38:203–207, 1991.
10. Tuppurainen N, Hallman M: Prevention of neonatal group B streptococcal disease: Intrapartum detection and chemoprophylaxis of heavily colonized parturients. Obstet Gynecol 73:583–587, 1989.
11. Boyer KM, Gotoff SP: Prevention of early-onset neonatal group B streptococcal disease with selective intrapartum prophylaxis. N Engl J Med 314:1665–1669, 1986.
12. Morales WJ, Lim D: Reduction of group B streptococcal maternal and neonatal infections in preterm pregnancies with premature rupture of membranes through a rapid identification test. Am J Obstet Gynecol 157:13–16, 1987.
13. American College of Obstetricians and Gynecologists (ACOG): Group B Streptococcal Infections in Pregnancy. ACOG Technical Bulletin No. 170. Washington, DC: ACOG, 1992.
14. American Academy of Pediatrics (AAP): Committee of Infectious Diseases and Committee on Fetus and Newborn. Guidelines for prevention of group B streptococcal (GBS) infection by chemoprophylaxis. Pediatrics 90: 775–778, 1992.
15. McDuffie RS, McGregor JA, Gibbs RS: Adverse perinatal outcome and resistant Enterobacteriaceae after antibiotic usage for premature rupture of the membranes and group B streptococcus carriage. Obstet Gynecol 82: 487–489, 1994.
16. Gonik B, Shannon RL, Shawar R, Costner M, Seibel M: Why patients fail antibiotic prophylaxis at cesarean delivery: Histologic evidence for incipient infection. Obstet Gynecol 79:179–184, 1992.
17. Watts DH, Hillier SH, Eschenbach DA: Upper genital tract isolates at delivery as predictors of post-cesarean infections among women receiving antibiotic prophylaxis. Obstet Gynecol 77:287–292, 1991.
18. Amstey MS, Gibbs RS: Is penicillin G a better choice than ampicillin for prophylaxis of neonatal group B streptococcal infections? Obstet Gynecol 84:1058–1059, 1994.
